# Unintended retention of a ruptured radiopaque thread extending from the corner of a gauze during laparoscopy

**DOI:** 10.1186/s13037-019-0209-1

**Published:** 2019-09-04

**Authors:** Yoshiaki Oshima, Osamu Yamamoto, Akihiro Otsuki, Saori Tokunaga, Keiichiro Ueda, Yoshimi Inagaki

**Affiliations:** 1 Department of Anesthesiology, Yonago Medical Center, 4-17-1 Kuzumo, Yonago, Tottori, 683-0006 Japan; 2 Department of Gastrointestinal Surgery, Yonago Medical Center, 4-17-1 Kuzumo, Yonago, Tottori, 683-0006 Japan; 3 Division of Anesthesiology and Critical Care Medicine, Department of Surgery, Tottori University Faculty of Medicine, 36-1 Nishi-cho, Yonago, Tottori, 683-8504 Japan

**Keywords:** Laparoscopy, Retention of gauze, Radiopaque thread, Gauze count, Electric scalpel

## Abstract

Small gauze is used in laparoscopy; therefore, retention of gauze can occur. We experienced a case of retention of a radiopaque thread that ruptured from a piece of gauze and moved into the peritoneum during a scheduled laparoscopy. The patient was a 65-year-old woman who underwent laparoscopic-assisted transverse colon resection for transverse colon cancer. A commercial gauze commonly used for laparoscopy was used during the surgery. To more easily identify the gauze during surgery, radiopaque threads extending up to 3.0 cm from the two diagonal corners of the gauze body were attached. After wound closure, radiography showed a radiopaque thread-like substance in the abdomen. Minor laparotomy was performed, and part of the radiopaque thread was discovered. On postoperative day 22, the patient was in remission and discharged.

**Letter to the Editor**

The frequency of unintended retention of gauze during surgery is 1 in 8300 cases [1], and the abdomen and pelvis are the most common anatomic sites of retention, accounting for 50.2% of such occurrences [2]. In patients with unintended retention of gauze, the gauze count was judged as correct in 86% of the cases [2]. Small gauze is used in laparoscopy, and therefore, unintentional retention of gauze can occur. The frequency of unintended retention of gauze has not yet been reported. We experienced a case of retention of a radiopaque thread that ruptured from the corner of a gauze and moved into the peritoneum during a scheduled laparoscopy.

The patient was a 65-year-old woman with a height of 151 cm and weight of 39 kg. At the age of 53, she underwent laparoscopic-assisted transverse colon resection due to transverse colon cancer. Surgery time was 3 h 18 min, and the volume of perioperative blood loss was 15 ml. Gauze count was performed four times: at the time of preparing the surgical instruments, immediately before the start of surgery, at the start of wound closure, and at the time of skin suture. At all times, the gauze count was found to be correct. The surgery was completed uneventfully. After wound closure, abdominal radiography was performed, and a radiopaque thread-like substance was found in the left side of the abdomen (Fig. 1). When all pieces of gauze used in the procedure were examined, one was found to have a radiopaque thread that had shortened due to rupture (Fig. 2a, bottom). The gauze itself had some burn marks on it (Fig. 2b); the end of the thread also had evidence of being burnt by an electric scalpel (Fig. 2c). Although it seemed that only the thread that ruptured from the corner of the gauze entered the peritoneum, the possibility of retention of the gauze itself could not be completely ruled out. Minor laparotomy was performed to look for the retained object. The part of the radiopaque thread that had ruptured from the gauze was discovered under fluoroscopic guidance (Fig. 2c), confirming that the gauze itself was absent during wound closure. On postoperative day 22, the patient was in remission and discharged. She has progressed uneventfully till the time of writing this correspondence.

**Fig. 1:**
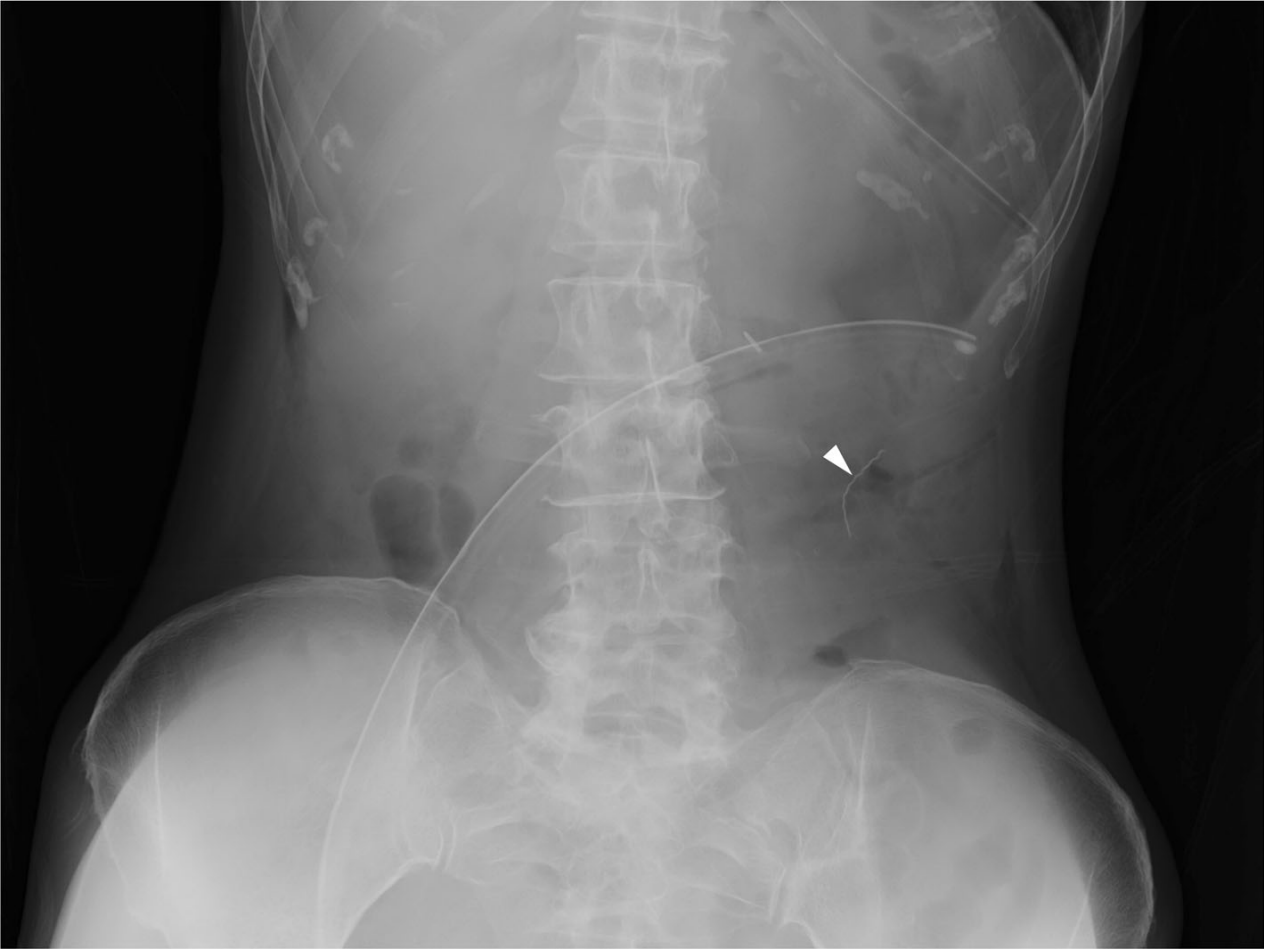
An X-ray image of the abdomen showed a radiopaque thread-like substance retained in the left part of the abdomen (arrowhead)

**Fig. 2:**
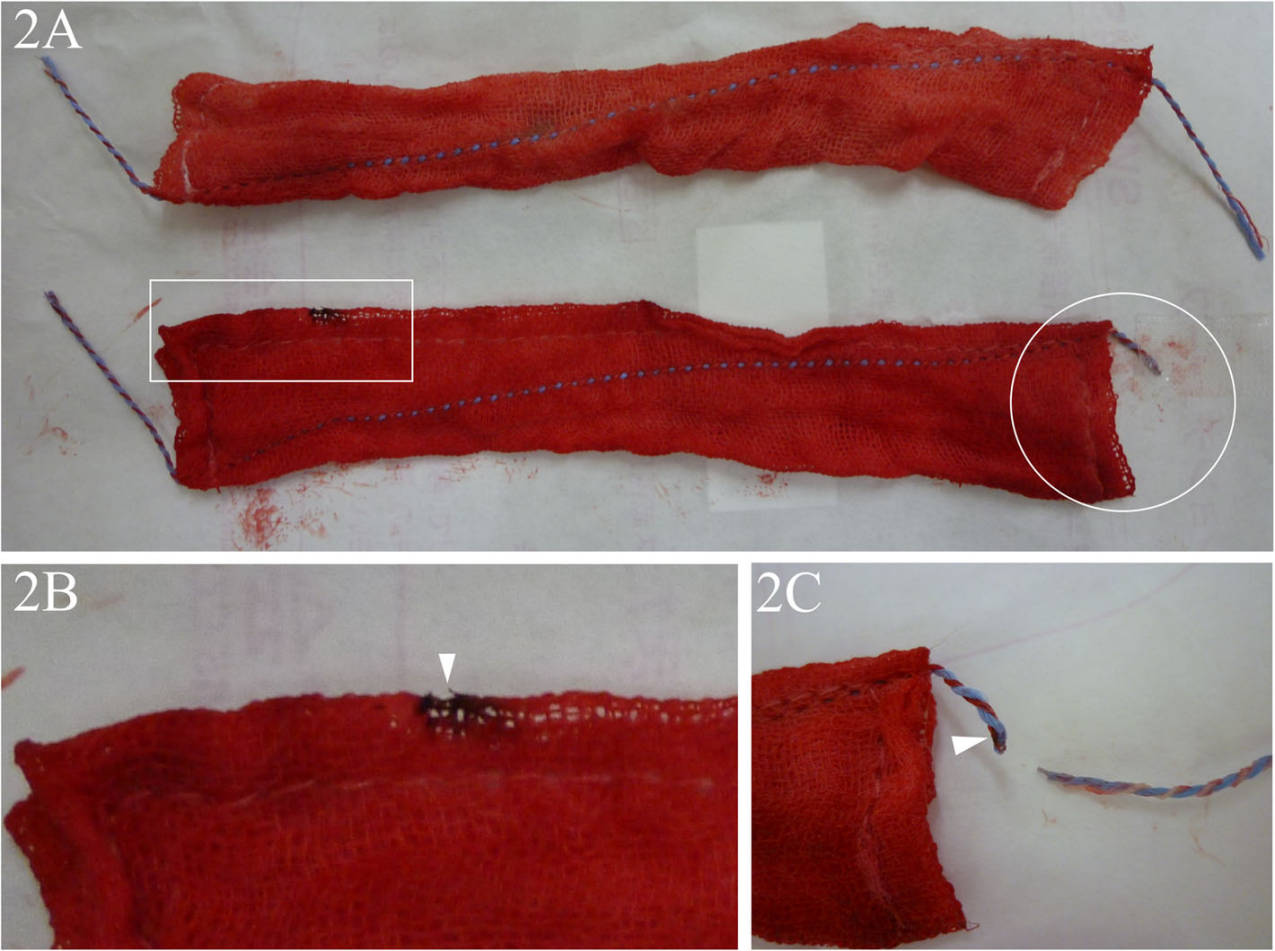
**a.** The upper panel shows regular gauze with the extending threads intact after its use. The lower panel shows gauze from which the extending thread ruptured at its midpoint during its use. **b**. A portion of the gauze body (enlarged image of the area in the white rectangle in **a**) has a burnt mark (arrowhead). **c**. The end of the thread remaining on the gauze (enlarged image of the area in the white circle in **a**) and the portion of the thread that entered the peritoneum are shown. The end of the remnant thread extending from the corner of the gauze has a burnt mark (arrowhead)

The gauze (Trox, Osaki Medical, Nagoya, Japan) used during surgery has been commercialised for laparoscopy. The body of the gauze is made of cotton; it is thin and long, with dimensions of 3.0 × 15.0 cm to enable its passage through a port (Fig. 2). As the lumen of the port is narrow, the corners of the gauze are compressed between forceps; the long axis of the gauze and that of the port are matched, and the gauze is then pulled out from the peritoneum through the port. For easy identification of the gauze during surgery and easily identifying the corners of the gauze, radiopaque threads extending up to 3.0 cm from the two diagonal corners of the gauze body are attached (Fig. 2a, top). A similar radiopaque thread is sewn diagonally through the gauze body (Fig. 2). The thread is made of polypropylene and polyester and becomes radiopaque when soaked in barium sulphate. Polypropylene and polyester per se are inactive. The maker put a precaution in the catalogue that direct contact of the extending radiopaque thread with an electric scalpel or direct compression with forceps should be avoided when using the gauze. The maker also produces gauze (Trox II) of the same size that has no radiopaque extension thread. We are presently using Trox II in laparoscopy at our hospital.

## Data Availability

The datasets used and/or analysed during the current study are available from the corresponding author on reasonable request.
